# Single Jejunum Metastasis from Breast Cancer Arising Twelve Years after the Initial Treatment

**DOI:** 10.1155/2016/8594652

**Published:** 2016-10-03

**Authors:** Cláudia Paiva, José Garcia, Cristina Silva, Alexandra Araújo, António Araújo, Marisa D. Santos

**Affiliations:** ^1^Department of Surgery, Digestive Surgery Service, Centro Hospitalar do Porto, Largo Professor Abel Salazar, 4099-003 Porto, Portugal; ^2^Department of Pathology, Pathological Anatomy Service, Centro Hospitalar do Porto, Largo Professor Abel Salazar, 4099-003 Porto, Portugal; ^3^Service of Medical Oncology, Centro Hospitalar do Porto, Largo Professor Abel Salazar, 4099-003 Porto, Portugal

## Abstract

Metastatic involvement of gastrointestinal tract from breast cancer is a rare event. We report the case of a 61-year-old woman presenting with bowel obstruction, related to metastasis of a primary breast cancer she had 12 years earlier (a triple-negative invasive ductal carcinoma treated with surgery and chemotherapy). Bowel obstruction was caused by a 20-centimeter tumor in the jejunum, involving also the transverse colon. The patient underwent en bloc resection of tumor with jejunum and transverse bowel segment and received adjuvant chemotherapy with carboplatin and paclitaxel. Twenty months later, she was alive without disease recurrence.

## 1. Introduction

Breast cancer is the most common malignancy in women and the second main cause of cancer-related deaths [1]. Approximately 5% to 10% of breast cancers are metastatic at diagnosis, and 20–50% of patients will develop metastatic disease during their lifetime [2], but the incidence of subclinical distant metastases is probably higher, as shown by autopsy series [3].

Metastases of breast cancer are commonly found in bone, liver, lungs, and brain. It rarely metastasizes to the gastrointestinal tract (GIT), with just a few cases of such metastases reported in the literature [4]. Although breast cancer is one of the few tumors metastasizing to the GIT, along with melanoma, ovarian cancer, and bladder cancer [5], this is still a rare event [6].

Metastatic patterns of invasive lobular carcinoma (LC) and invasive ductal carcinoma (DC) usually differ, and LC is more prone to metastasize to the GIT [7]. Nowadays, with longer survival due to advances in treatment, metastatic spread may occur several years after primary surgical and systemic treatment. In this regard, we report the case of a patient with a single breast cancer metastasis to the jejunum, presenting with bowel obstruction 12 years after her initial diagnosis.

## 2. Presentation of Case

This is a 61-year-old woman with previous history of locally advanced left breast cancer diagnosed in 2002. She had four cycles of neoadjuvant chemotherapy with doxorubicin (60 mg/m^2^) and cyclophosphamide (600 mg/m^2^) between September 27 and November 29, followed by lumpectomy with ipsilateral axillary lymph node dissection in December 26, 2002. Histopathological examination of the tumor revealed invasive ductal carcinoma (IDC), histologic grade III, classified as ypT2N3cM0, stage IIIC. Immunohistochemical staining was negative for estrogen receptor (ER), progesterone receptor (PgR), and human epidermal growth factor receptor 2 (HER2). She had adjuvant chemotherapy with four cycles of paclitaxel (80 mg/m^2^) between January 31 and April 04, 2003, and adjuvant radiotherapy 50 Gy to her left breast, axillary, and supraclavicular areas and an additional 10 Gy boost to the primary tumor site, finished on June 28, 2003. She has been disease-free for the last 12 years.

**Figure 1:**
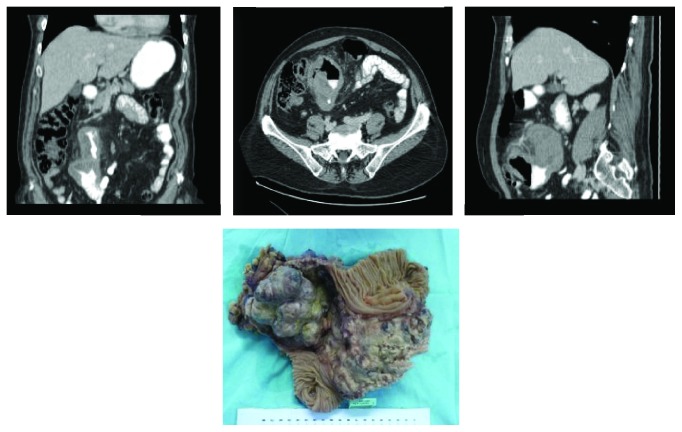
Tumor aspects in CT scan and in resected specimen.

This woman presented to our Emergency Department in July 31, 2014, with a 2 months' history of colicky abdominal pain after meals, worsening constipation, abdominal distension, unintentional weight loss of 5 Kg, and tiredness. The physical examination revealed a palpable mass at the right abdominal quadrants. Laboratory tests showed anaemia (haemoglobin count of 8.4 g/dL). A computed tomography (CT) scan was performed and revealed a 20-centimeter mass in the jejunum, which also involved the transverse colon (Figure 1). She was proposed to elective surgery in the same hospital admission. Laparotomy (August 6, 2014) confirmed the jejunal mass; resection of the jejunal tumor along with a segment of transverse colon was performed. The postoperative period was uneventful and the patient was discharged home on the 8th postoperative day.

**Figure 2:**
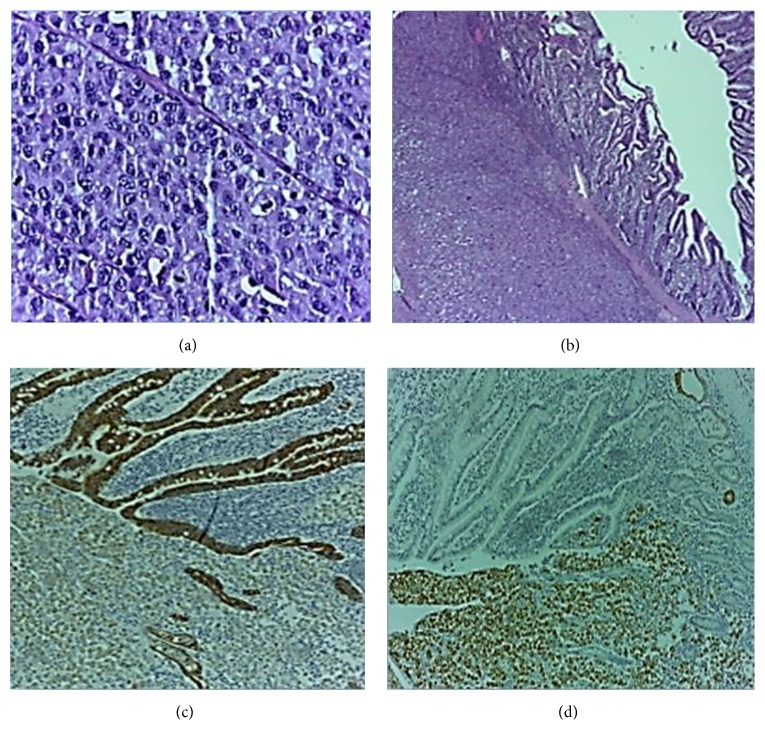
Histologic and IHC study: (a) and (b) indifferenciated carcinoma (hematoxylin and eosin stain) and (c) and (d) positivity for CAM 5.2 and cytokeratin 7.

Histopathological examination of the tumor revealed an undifferentiated carcinoma (T3N0Mx). The lack of dysplasia and atypia of the intestinal epithelium and the normal glands surrounding the malignant cells favored diagnosis of a metastatic lesion. Immunohistochemistry (IHC) performed on the specimen was positive for cytokeratin 7 (CK7) and CAM 5.2 (Figure 2) and negative for ER, PgR, and HER2 receptors, cytokeratin-20 (CK20), and gross cystic disease fluid protein-15 (GCDFP-15). Histologic slides revision was performed in other institutions with agreeing results: IHC was negative for mammaglobin and GCDFP-15. GATA3 expression is not performed in both institutions. Notwithstanding this, IHC along with morphological aspects of the tumor were consistent with breast cancer metastases. Based on these findings and the clinical history, we concluded this was a jejunal metastasis from breast carcinoma.

The patient started on adjuvant chemotherapy in October 2, 2014, with 6 cycles of paclitaxel (175 mg/m^2^) followed by carboplatin (AUC 5–685 mg), and ended it in January 27, 2015. She is disease-free for 20 months now.

## 3. Discussion

Metastases of breast cancer are commonly found in bone, liver, lungs, and brain. Gastrointestinal (GI) metastases from breast cancer are rare in clinical practice, with less than 1% of breast cancer having GI metastases [7, 8]. Isolated GI metastases without metastases to other organs are even rarer. In the review by McLemore et al. [6], out of 12,001 cases of metastatic breast cancer, only 23 cases had GI metastases alone (0.2%). Autopsy series show that the occurrence of GI metastases from breast cancer is more common, than clinically suspected, reaching 30% of cases in some studies [3, 5]. However, most of these metastases are asymptomatic and, therefore, not detected during follow-up and with less clinical relevance. When looking at breast cancer metastases to GI tract, the patterns of metastatic spread are quite variable in the literature, with small bowel being affected in 9–28% of cases with GI metastases. A paper published in 2012 by Ambroggi et al. analyzed the sites of GI breast cancer metastases. They reviewed the literature and found the most common site of GI metastases of breast cancer was the stomach (60%) and followed by esophagus (12%), colon (11%), small bowel (8%), and rectum (7%) [8].

Metastatic patterns vary according to histological types. Although LC accounts for only 5–10% of invasive breast carcinoma, this histological subtype is the one more frequently associated with metastases to the GIT [6–8]. Harris et al. published a landmark study comparing metastatic pattern of LC and DC [3]. He found that, in 76 cases of metastatic DC, only 3 had intestinal metastases. He also found that LC was prone to metastasize in a more “diffuse” fashion, with multiple tiny nodules that became confluent, while DC metastases formed nodular masses. Our patient had a metastasis of DC to the small bowel, which is a rare event but has been described previously [6, 8–11].

With regard to time between breast cancer and GI metastases diagnosis, most cases usually occur in the early years after primary cancer diagnosis. Switzer et al. [12] reported a median interval of 4 years between breast cancer and GI metastases and McLemore et al. [6] found a median of 7 years. Our patient presented with a metastasis occurring 12 years after her primary breast cancer diagnosis. This is uncommon but longer time periods have been published in the literature, with GI metastases presenting as late as 30 years [13].

The correct diagnosis on this kind of clinical case can be a hard challenge. First is the rarity of the condition that leads to a low index of suspicion. Second, they can occur many years after the original disease, especially with longer survival of patients. Third, they have a nonspecific clinical presentation: symptoms depend on localization and range widely from a patient being relatively asymptomatic [9] to complaining of vague nausea, abdominal pain [12], dysphagia [4], or more alarming symptoms such as bleeding [14], bowel obstruction [10, 15], perforation [16], or even appendicitis [17]. However, even if GI metastases of breast cancer are rare, this diagnostic hypothesis should be considered in any woman presenting with a GI tumor and a history of breast cancer (regardless of how remote).

Another issue is the pathological diagnosis. In fact, distinguishing primary from metastatic GI tumors can be difficult. Features typically used to make this distinction include gross configuration of the tumor [4], careful evaluation of the morphology of the lesion on histopathological analysis, and, when possible, comparative review of slides of prior cancer. All of those are as important as the clinical history in diagnosis. Usually, morphology on slides is similar enough to allow a diagnosis of metastatic breast cancer based on comparative histologic review alone [18]. In IHC study, carcinomas of breast origin are usually positive for CK7 and negative for CK20 (CK7+/CK20−), contrary to low GI tumors that are almost always CK20 positive [19]. In the present case the primary tumor and the metastasis are poorly differentiated, making comparative histopathological study difficult and renders IHC study poor diagnostic value. In fact, IHC alone should not be used, especially in metastases of poorly differentiated cancers, which tend to lose tissue-specific gene expression compared to those of the primary tumor. Despite these limitations, our patient's primary breast cancer and the metastasis diagnosed 12 years later are both CK7+/CK20−. Although changes in receptor status over the course of disease have been described, in most cases there is concordance between primary tumors and matching metastasis [20]. This profile is seen in other tumors, such as lung or the gynecologic malignancies; however, careful attention to the clinical history and the morphologic findings of the tumor favored a breast origin. In our case the GCDFP-15 and mammaglobin were both negative. This is an expected result, since in triple-negative breast cancer (TNBC) GCDFP-15 is infrequently expressed when compared with other breast cancer subtypes [21]. Also, diagnostic sensitivity of GCDFP-15 is only moderate with reported rates of expression in metastatic breast cancer ranging from 11% to 71% [21]. Mammaglobin is more sensitive than GCDFP-15 for breast origin but less specific, being negative in 23–52% of locally recurrent and metastatic breast carcinomas [21]. GATA3 seems to be particularly useful as a marker for metastatic breast carcinoma, especially triple-negative and metaplastic carcinomas, which lack specific markers of mammary origin [22], but is not available in both institutions. Anyway, the IHC results must be integrated with the morphologic and clinical features to arrive at the correct diagnosis. In this case, the lack of dysplasia or atypia of the intestinal epithelium and the glands surrounding the malignant cells was an important factor in making the diagnosis of a metastatic lesion more likely.

So, our diagnosis of breast cancer metastasis was based on the combination of available clinical history, histology, and IHC. In this case, no isolated tissue marker was entirely specific or sensitive, but their integration with clinical history and morphological findings allowed us to make the diagnosis.

Regarding treatment options for metastatic breast cancer patients, options are targeted therapies (endocrine therapy or molecularly targeted agents such as trastuzumab or lapatinib) or systemic chemotherapy. Choice of treatment will be dictated by the IHC profile and patient's symptoms. Patients with TNBC lack known drug targets (ER, PgR, and HER2); hence they are treated with chemotherapy [6, 8, 23]. Surgery and radiotherapy are usually used with palliative intent, for symptom relief [6]. Surgery is mainly indicated to treat complications such as bleeding, perforation, or obstruction [6, 10, 15]. In selected cases, surgery may increase survival [24]. Survival rate of patients who undergo treatment averages 24 months and is being improved by new systemic therapies [25]. In this case surgery was indicated due to intestinal obstruction and adjuvant chemotherapy (carboplatin and paclitaxel) was selected according to the diagnosis of triple-negative breast cancer metastasis. Currently, 20 months into follow-up, the patient is asymptomatic and free of known disease recurrence.

This manuscript alerts clinicians to the importance of being attentive to gastrointestinal symptoms in breast cancer patients, no matter how long ago the breast cancer was diagnosed, as they can be the first sign of GI tract metastases. This can lead to a correct diagnosis, which can allow the patient to have the necessary treatment. The relevance of this case is also related to the fact that diagnosis of breast cancer metastases to GI tract more than 5 years after initial cancer treatment can be a challenge, especially if it is a single metastasis to GI tract of a TNBC, in which case the IHC alone may not be enough for a diagnosis. It was the combination of available clinical history, histology, and IHC that allowed for a correct diagnosis.

## 4. Conclusion

Although rare, GIT breast cancer metastases can occur. It is important for physicians to keep in mind that clinical presentation is random and nonspecific, and that GI complaints can occur years after initial disease. It should also be remembered that this pattern of metastatic spread is not limited to LC but may also be seen with the commoner DC. Diagnosis should be quick and prompt intervention guaranteed. Patients should be offered surgery in cases of complication or for symptomatic relief.
